# Why Secondary Schools Do Not Implement Far-Reaching Smoke-Free Policies: Exploring Deep Core, Policy Core, and Secondary Beliefs of School Staff in the Netherlands

**DOI:** 10.1007/s12529-019-09818-y

**Published:** 2019-10-28

**Authors:** Michael Schreuders, Bas van den Putte, SILNE-R consortium, Anton E Kunst

**Affiliations:** 1 Department of Public Health, Amsterdam Public Health Institute, Amsterdam UMC, University of Amsterdam, Amsterdam, The Netherlands; 2 Faculty of Social Sciences, Unit of Health Sciences, Tampere University, Tampere, Finland; 3 Faculty of Social and Behavioural Sciences, Amsterdam Public Health Institute, University of Amsterdam, Amsterdam, The Netherlands

**Keywords:** Adolescents, Schools, Smoking, Smoke-free, Tobacco control, Advocacy coalition framework

## Abstract

**Background:**

Secondary schools that implement smoke-free policies are confronted with students who start smoking outside their premises. One solution is to complement smoke-free policies with prohibitions for all students to leave the school area during school hours, technically making school hours a smoke-free period. However, there are strikingly few Dutch secondary schools that implement this approach. This study explores why staff members in the Netherlands decide not to implement smoke-free school hours for all students.

**Method:**

We interviewed 13 staff members, with different functions, from four secondary schools. The analysis was informed by the Advocacy Coalition Framework (ACF) to delve into the values, rationales, and assumptions of staff with the aim to identify deep core, policy core, and secondary beliefs.

**Results:**

We identified six beliefs. Two deep core beliefs are that schools should provide adolescents the freedom to learn how to responsibly use their personal autonomy and that schools should only interfere if adolescents endanger or bother others. Three policy core beliefs identified included the following: that smoking is not a pressing issue for schools to deal with; that schools should demarcate their jurisdiction to intervene in adolescents’ lives in time, space, and precise risk behavior; and that implementing smoke-free school hours would interfere with maintaining positive student-staff relationships. One secondary belief identified was that smoke-free school hours would be impossible to enforce consistently.

**Conclusion:**

This paper was the first to demonstrate the many beliefs explaining why schools refrain from voluntary implementing far-reaching smoke-free policies.

## Introduction

Adolescent smoking in European countries remains an issue [1, 2]. Most European governments, therefore, increasingly compel secondary schools to implement smoke-free school policies (SFSPs) that prohibit all from smoking both in school buildings and on the premises of schools. Scientific evidence indeed suggests that this comprehensive approach to SFSPs is more effective than SFSPs that allow smoking in restricted areas and/or exempt certain individuals [3].

However, the implementation of comprehensive SFSPs does not necessarily stop adolescents from smoking during school hours. Adolescents may simply circumvent comprehensive SFSPs by relocating their smoking to sites outside of the school’s premises [4]. This relocation, as a side-effect of SFSPs, brings about its own issues. Firstly, adolescent smoking just outside the school premises (e.g., at the entrance) may increase the visibility of smoking for non-smokers [5], which has been associated with increases in the susceptibility of non-smokers to initiate smoking and the actual initiation of smoking [6, 7]. Secondly, the adolescents at risk of smoking may band together outside the premises and develop pro-smoking social meanings [8], such as using smoking as a means to challenge adult opposition [9] and isolate themselves from others [10].

Complementary to comprehensive SFSPs, some national (e.g., Finland) and regional (e.g., Flanders) governments compel secondary schools to prohibit all adolescents from leaving the school area during school hours. This combination of policies technically makes the school hours an entirely smoke-free period. To our knowledge, the only study so far that compared SFSPs with and without prohibitions to leave the premises of schools took place in Finland. This study indeed found that the former was associated with lower smoking rates [11]. Currently, the Danish PROVE-DK research project is studying the effectiveness of implementing smoke-free school hours on the smoking behaviors of adolescents enrolled in vocational schools.

Importantly, while most European governments have not adopted legislation on smoke-free school hours, this does not prohibit schools from implementing this rule themselves. Schools indeed have the authority to prohibit adolescents from leaving the school premises. Nonetheless, to our understanding, there are very few schools that choose to do so in order to implement smoke-free school hours for all adolescents. Contemporary scientific literature does not sufficiently help to unpack why this is the case. A first reason impeding research into this area is the fact that studies have commonly focused on the adoption of SFSPs in schools that continued to allow smoking outside the school premises [12, 13]. A second reason is that studies on the adoption of health programs/policies in schools have largely focused on the identification of mostly external facilitators and barriers. As such, these studies have not fully explored the extent to which staff members’ values, rationales, and assumptions may explain the non-adoption of smoke-free school hours [14, 15].

Gaining a more in-depth understanding on this issue is important for two reasons. First, new insights can inform discussions on how far schools should, can, and would like to go in addressing adolescent smoking. There may be well-founded reasons explaining why staff members, at least currently, refrain from adopting a stricter stance on adolescent smoking. Second, new insights may allow tobacco control advocates to better convince and support schools to increase their efforts to address the problem of adolescent smoking during school hours.

Thus, the aim of this paper was to explore the considerations of staff members in Dutch secondary schools regarding the implementation of smoke-free measures and reasons for deciding not to make school hours a smoke-free period. The Netherlands is an interesting context for this investigation due to the fact that there is currently no legal obligation for schools to implement health promotion policies and consequently, schools may choose their own priorities [16]. Box 1 provides additional background information on the Netherlands.

Box 1

**Table:** 

Smoking prevalence: HBSC survey data from 2017 on 12–16 years old adolescents showed that 2.1% smokes daily and 5.7% smokes monthly [17]. National survey data from 2017 on adults showed that 17.2% smokes daily and 5.9% smokes occasionally [18]. Both adolescent and adult smoking is two to three times more prevalent among lower than higher educated individuals. National tobacco control environment: Tobacco control in the Netherlands had a slow start, but in 2016 ranks above-average in comparison to other European countries [19]. Advocacy for more tobacco control is increasing in strength and receiving increasing media coverage. Currently, there is a strong national coalition striving for a smoke-free generation, and the government has involved many stakeholders in an agreement to achieve this goal by 2040. The legal age of sales for tobacco, alcohol, and marijuana products is set at 18 years of age, but the use of these substances by minors is not illegal. Advertising is prohibited and smoking is not allowed in work places and the hospitality industry. Public support for tobacco control: The general public increasingly supports existing tobacco control policies as well as the adoption of future tobacco control policies [20]. This support for future policies is particularly strong when policies are framed to protect children [21]. Smoke-free school policies: In 2017, the law only prohibits smoking inside the school buildings, with the exception of ventilated rooms. Nevertheless, at the time of study, more than half the secondary schools had implemented rules that did not allow any smoking inside the school building and at the premises. Dutch national parliament adopted an amendment of the Tobacco Law in February 2016, compelling all secondary schools to implement comprehensive SFSPs before August 2020 (i.e., no smoking inside the building and at the premises). Laws will not compel schools to prohibit anyone from leaving the school premises. School-based smoking education: School-based education about smoking is part of the curriculum, predominantly as part of the subjects “biology” and “care.” Schools may voluntarily choose to organize additional information campaigns, which is stimulated and facilitated by the national healthy school approach and the regional public health services.	

## Theoretical Framework

The Advocacy Coalition Framework (ACF) is a policy process framework that assists in describing policy-making environments and explaining policy change [22]. The ACF is praised for integrating top-down and bottom-up policy change processes into a single framework. This paper builds on one of its key insights: the likelihood for stakeholders to use their power to adopt new and adapt existing policies depends largely on their belief systems. A belief system consists of three interdependent types of beliefs that determine staff members’ opinion about adopting policies that would make the school hours a smoke-free period.

The first type are deep core beliefs. These beliefs are based on societal values that are relevant to a specific policy. Scientific literature suggests that in the Netherlands, like in most Western cultural contexts, staff members’ deep core beliefs likely build on two possibly contradictory positions: first, the position that schools should respect adolescents’ freedom to choose to smoke cigarettes [23, 24], relating to values of individualism and autonomy [25]; second, the position that schools should protect vulnerable individuals against the harms of cigarette smoking and exposure to secondhand smoke [26], relating to values of solidarity and social responsibility.

The second type of beliefs are policy core beliefs*.* These beliefs are based on views about the severity of the problem, who is responsible for resolving the problem and what is the preferred mode to resolve a problem [22]. Scientific literature about schools suggests that implementation of anti-smoking policies is influenced by the views of staff members on a number of areas, such as whether smoking during school hours is a problem, to what extent schools should take the responsibility to deal with smoking, and under what conditions the school should complement the common educative measures with more restrictive measures [15, 27, 28].

The third type are secondary beliefs*.* These beliefs are based on views about the context-specific feasibility of implementing a policy. Scientific literature provides many insights into what anticipated feasibility problems could underlie secondary beliefs because these are often identified in studies about barriers for the implementation of school health programs. Recurring examples are concerns about finances, resistance from smoking staff members, the absence of government policy, increases in work pressure, and the difficulties to monitor rules [12, 29, 30].

This study therefore identified Dutch school staff members’ deep core, policy core, and secondary beliefs explaining why they decide not to implement smoke-free school hours for all adolescents.

## Methods

### Research Design

This study is part of the European SILNE-R project which aims to develop insights for enhancing the impact of tobacco control measures on youth smoking (http://silne-r.ensp.org/). Researchers in this project collected qualitative and quantitative data of school staff members and students from end-2016 to mid-2017. For this paper, we performed a secondary analysis on qualitative interview transcripts with school staff members from the city of Amersfoort, the Netherlands. Amersfoort was chosen as it reflects the national average in terms of demography, unemployment rate, income, and proportion of migrants [31]. We also analyzed the quantitative survey data of adolescents, but only to provide background information about the smoking prevalence per school.

### Sample

Six secondary schools in Amersfoort participated in quantitative data collection. There were no strict selection criteria, except that the final sample of schools should represent all educational levels. The Dutch educational system is tiered and separates adolescents into low (preparation for tertiary vocational school), middle (preparation for college), and high (preparation for university) levels. The first author—whom collaborated in the SILNE-R project—prepared a selection of schools for the purpose of this study, proposing four of these six participating schools based on their differing educational levels. This number of schools was a priori decided by the SILNE-R research consortium before data collection. Representatives of each school were contacted in late-2016 to request their agreement to participate in the qualitative data collection among school staff. All four schools approved.

The first author recruited potential interviewees in person, joined by the contact person at each school, in order to ensure the selection of participants with sufficient experience with smoke-free school policies (e.g., involved in enforcement or decision-making). We purposefully recruited staff members with different functions: principals, teachers, and persons in a supportive role that frequently are in contact with smokers (e.g., janitor, receptionist). All staff members approached agreed to participate. Most interviewees did not have any substantial contact with the interviewer until the interview. In total, 13 interviews were held, three interviews in three schools each, and four interviews in one school.

Table 1 presents the characteristics of the four schools, including information about the educational level, number of students, percentage of weekly smoking students, and rules on smoking and leaving the school premises. Schools 1 and 3 did not allow any smoking on the premises. Schools 2 allowed smoking on the premises in a designated smoking area for adolescents from the third grade (typically 14–15 years in Dutch system) and later grades (up to grade six, 17–18 years). School 4 had a smoking area for adolescents from the fourth (15–16 years) grade onwards. Schools 1, 2, and 3 allowed adolescents from the third and later grades to leave the school premises during school hours. School 4 allowed this only from the fourth grade onwards. Adolescents who left the premises were not prohibited from smoking just outside the school premises.

**Table 1:** Overview of the schools and their characteristics

School	Educational level	Student weekly smoking (%)	Number of students	Permission to smoke for students
1	Middle	6.6	750	3rd graders and above, outside the premises
2	Middle and high	7.0	1000	3rd graders and above, in a designated area
3	Low	21.5	350	3rd graders and above, outside the premises
4	Low	18.8	250	4th graders and above, in a designated area

Table 2 presents an overview of the participants and information about their function, smoking status, age, and sex.

**Table 2:** Overview of participants

School	Code	Function	Smoker	Age	Gender
1	1T	Teacher	No	31	Female
1	1P	Principal	No	58	Female
1	1S	Supportive	Yes	47	Male
1	1ST	Senior teacher	No	34	Male
2	2S	Supportive	No	61	Female
2	2P	Principal	No	50	Male
2	2T	Teacher	Yes	61	Male
3	3P	Principal	No	52	Female
3	3T	Teacher	No	36	Female
3	3S	Supportive	No	54	Male
4	4T	Teacher	No	35	Male
4	4S	Supportive	No	46	Female
4	4P	Principal	No	61	Female

### Data Collection

The interviews followed a semi-structured interview guide that was pilot tested at school 1. The interviews were held at the beginning of 2017 by the first author; a male, Dutch 27-year old (MSc, PhD candidate) with substantial training and experience in qualitative data collection and analysis. The interviews took place in a quiet room in each school with no others present, were held in Dutch, and lasted between 45 and 60 min.

The interviewer first introduced himself, explained the purpose of the interview and participants’ right to confidentiality in scientific research, and asked the interviewee for their written consent and approval to voice-record the interview. Each interview began by asking staff members to talk about the school, their tasks in the school, and how long they have been working for the school. Then, participants were asked about the current school rules that apply to smoking and the adoption process that led to the current rules. They were also probed into explaining why the school does not make school hours a smoke-free period for all adolescents. The interviewer avoided as far as possible that participants would feel judged when asking why they choose *not* to do something. Strategies to do so included gradually steering the interview into these topics as well as emphasizing that the interviewer simply was interested in understanding why (e.g., “Now I am going to play devil’s advocate, but why is it that…”). After sufficiently exploring the adoption of the current school smoking rules, the interview concluded by the interviewer posing the question: what would facilitate schools in becoming entirely smoke-free in the future.

The participants were asked following the interview to fill out a short questionnaire about their age, gender, formal position in school, and smoking behavior. The first author made field notes immediately following the interviews. The impression was that most participants responded freely to the interviewers’ questions, but that participants’ initial responses could become more nuanced as the interview progressed.

### Data Analysis

Each interview was transcribed verbatim in Dutch using the voice-recording and translated into English. These English transcripts were then uploaded in MAXQDA12 (VERBI GmbH, Berlin, Germany). This is a software package for electronically organizing and coding qualitative data. We applied a thematic analysis [32], using a preliminary coding scheme based on the ACF belief system, distinguishing between deep core, policy core, and secondary beliefs, while also inductively analyzing the raw data.

The first author started the analysis of the raw data by reading and re-reading all transcripts, coding any piece of text that could explain why a school should or should not make school hours a smoke-free period, and categorizing similar codes into categories. To follow, this data was analyzed for themes throughout the categories and associated coded pieces of text. These were subsequently juxtaposed to each other and combined into coherent and distinct deep core, policy core, and secondary beliefs about the adoption of smoke-free school hours. The analytic steps after in-text coding and categorizing involved intensive collaboration between all authors, including the colleagues participating in the SILNE-R consortium, to construct, review, and interpret the themes with view to the original data and to establish and find consensus on the final beliefs. Agreement was reached among the authors on the six beliefs presented in the “Results” section.

## Results

We identified two deep core beliefs, three policy core beliefs, and one secondary belief. In the subsections that follow, each of these are presented and discussed.

### Deep Core Belief: Schools Should Provide Adolescents the Freedom to Learn How to Make Responsible Use of Their Personal Autonomy

Staff members argued that schools should take into account that there is a difference in the developmental needs between younger and older adolescents. The exact age demarcation between younger and older adolescents differed between the schools. This aside, the “general tendency that schools have” (3P) is to consider those from about the third grade (i.e., from 15 years of age) to be the older adolescents.

Schools should “keep an eye” (3P) on the younger adolescents by prohibiting them from leaving the school premises during school hours. This choice for being “rather protective of our [younger] students” (1P) is based on the belief that “they are not yet mature enough to make good use [behave responsibly] of the time they spend outside of the school” (1S). Schools therefore, want to “guarantee parents that their children are in the school [under supervision]” (2S).

The older adolescents, in contrast, are perceived as “wiser and smarter” (4T), become “more articulate,” and “start to investigate, explore and choose their own path” (4S). According to interviewees, schools should, therefore, offer older adolescents the opportunity to leave the school premises but simultaneously set “rules that provide them a clear-cut structure” (2P) in order to ensure they learn to make responsible use of the freedom to make independent choices.

[We] should give them a bit more freedom (…) but the rules clearly state that the moment you [the student] abuse that freedom, then we can tell you that you’re no longer allowed to leave the school premises during school hours (1ST).

Staff members, in this perspective, explicitly stated that life is more than the secondary school environment and that schools should therefore, “prepare them for the real-world” (1P). As such, smoking during school hours was framed as an available choice that the older adolescents themselves are responsible for.

You should give students [older adolescents] the freedom to decide what they’re going to do. And if that includes smoking, then, well, alright. So be it. But it’s still a choice that you’re making. You’re [as school] trying to make people aware of why they’re doing some things (…) We won’t say: “careful, you’re not allowed to do that.” Because you carry the responsibility of your own well-being (1S).

I don’t feel responsible for them [older adolescents]. They should just decide for themselves, and everyone wants to try... During my younger years, I also wanted to see what smoking was like (3S).

Nevertheless, staff members argued that schools should demotivate older adolescents from choosing to smoke by making the smoking spots “uninviting” (2S) and as “unattractive as possible” (4S). This approach—referred to as “demotivation policy” by some staff members—was reasoned to decrease adolescents’ smoking uptake by influencing the attractiveness of the choice to smoke, while still respecting older adolescents’ autonomy to choose to smoke.

### Deep Core Belief: Schools Should Interfere If Adolescents Endanger or Bother Others

Staff members did not consider adolescents’ smoking to be a problem as long as they were not endangering or bothering others.

It’s simple: smoking is not good for your health. But do I make a fuss about it? I don’t really make a fuss about it (…) what is important is that you’re not endangering others, that you’re not taking away their safety, and in my opinion that isn’t really the case with smoking (1ST).

Staff members discussed two ways in which smokers may bother others and framed these as strong motivations for schools to change the existing rules about where adolescents are allowed to smoke. Firstly, it was argued that smokers may bother non-smokers with secondhand smoke.

Yes, they came to me and said ‘sir, we can’t sit anywhere, because everyone is smoking everywhere’, and that was when I said that we had to do something about it (3S).

Secondly, smokers had a tendency to wander around and cause a nuisance in the residential areas surrounding the school. The interviewees argued that SFSPs make adolescents’ wandering worse. All schools, therefore, decided to allow students to smoke at or just outside the school premises because it decreases the nuisance that smokers cause to the surrounding area.

Last year, we created that [smoking] spot because we often had that third- fourth- or fifth-year students that were allowed to leave the school premises that would stand here on the pathway, and then a lot of people would no longer be able to walk past. (…) So then we said: ‘You know, we could offer them that structure and keep them in one spot’ (4T).

Two staff members from one school argued that the school is progressively (i.e., first the third year, a year later the fourth year, etc.) prohibiting all adolescents from leaving the school premises. The reason to do so was, however, not to further decrease smoking but to stop the nuisance that adolescents were still causing to the schools’ neighbors.

[We made this decision] because the students wander the streets and trouble our neighbors. They [the neighbors] tell us that ‘it’s great that you have such a smoking policy, but now they are smoking in front of our yards’ (3S).

### Policy Core Belief: Schools Should Prioritize the Pressing Health and Social Issues

Staff members did not consider further decreasing adolescent smoking during school hours as a school priority. According to interviewees, schools’ main priority is teaching the set curriculum (e.g., math, history, etc., but also some health-related topics), whereas health promotion per se only becomes a priority if specific behaviors are seen as an issue that is pressing enough. Schools dealt with the health or social issues that they—each school for their own reasons—considered most pressing.

I’m [principle of] a school. I must make sure that my staff, and that my main priority, namely education, is done well. And education does mean that I teach them about being healthy, but it doesn’t mean that I enforce a rule, which is difficult to enforce, so strictly that people who already have a ton of work to do [school staff], have even more work to do (2P).

If we as a school are as flexible as we are, then we’ll notice that if a problem would arise in some area, that we’d pick up on that and respond to that. For instance, we’ve noticed that with the current use of WhatsApp, that sexting [sending nude picture of oneself to others] is a much bigger issue in our school than smoking (1ST).

Staff members put forward two central arguments underpinning why smoking during school hours currently is not a pressing issue. Firstly, staff members argued that the attractiveness for adolescents to start smoking during school hours has decreased due to a major shift in the societal perception of smoking.

We still have smokers, but less than before, because the image of smokers has changed over time (…) if I look at the way it was 11 years ago when I started working here, and compare it to the way it is now, then I get the idea that people don’t find smoking as cool as they did before (4S).

In parallel to this decrease in attractiveness of smoking, SFSPs were argued to “work sufficiently well” (2P) in demotivating smoking as well as limiting the social interactions between younger adolescents and smokers.

Secondly, the current rules are sufficient to meet the expectations of parents and people living in the school area. Meeting these expectations was considered to be important because it “promotes the school” (4S) and therewith helps the school to attract new first-year students.

It is very strange that pupils who are not 18 are allowed to smoke during school hours… so… yah… why this additional step is not made… I think it has to do with the lack of urgency. We are implementing a smoking policy that works well, of course not perfect, but for the PR-picture it works well. That is obviously always a point (1T).

Staff members’ view thus was that schools should not prohibit the older adolescents from smoking during school hours because the measure does not stand in proportion to the problem and does not contribute to improving the schools’ image to the outside world.

### Policy Core Belief: Schools Should Demarcate Their Jurisdiction to Interfere in Adolescents’ Lives

Staff members explicitly demarcated their jurisdiction to interfere in adolescents’ lives in time, space, and the precise risk behavior, and stressed that such demarcation allows staff members to build relationships with students that are independent and complementary to adolescents’ parents. The school area was put forward as the natural place for schools to hold authority because the school is “responsible for everyone, and if something happens in school corridors” the school has “to talk to the students and fix it” (2P).

Parents who questioned the school smoking rules (i.e., parents who want to allow their child to smoke at the premises) were categorically refuted. According to interviewees, the parents should know what the schools’ rules are and by enrolling their child to a particular school means that they have to agree with the school rules.

I always think that these are our school rules and if you disagree, then take your kid and enroll them at a different school where those rules don’t exist, as it always happens over there. But it’s always ‘my daughter this, my daughter that’. And I think that ‘these are simply the school rules’. You signed up for that and you’re going to have to abide by it (3S).

Well, that our views differ, which is a reason for us to talk with the parents, but no matter what happens, our [smoking] policy will be enforced (4P).

The staff members, in contrast, also argued that schools have little to say about older adolescents’ choice to smoke outside the school area and any adolescents’ smoking after the school hours. The only ones who have such authority are the parents.

Then the next step is that you’d want adolescents to stop smoking, but that responsibility really lies with the parents. That’s where the next step should take place. Being a school, and you’ll notice this, as a school we do have the tendency to take on the role of the parents (…) you have to draw the line at some point (1ST).

Smoking therefore also was not something to bring up in a conversation with parents as long as adolescents adhere to the existing school rules.

If I’d do it [enforce school rules] outside of school hours too and tell them [parents]: ‘I’ve seen your son or daughter [smoking] last night at eight o’clock’, then these kids aren’t going to trust me anymore. Then they’re not going to negotiate with me anymore. I have to be there for them (4S).

In contrast, this physical and temporal demarcation of schools’ jurisdiction was less strong for the use of alcohol and drugs during school hours. Adolescents’ use of these substances during school hours was strictly forbidden and so the informing of parents was a more common practice.

Yes, sometimes we do [talk about smoking with parents]. If it’s a topic that comes up or if we’re in a conversation, but we are much quicker to point out the use of alcohol or drugs rather than smoking (3P).

### Policy Core Belief: Schools Should Be Able to Maintain Positive Relationships with Smokers

Staff members argued that maintaining a positive relationship with smokers is of key importance for adolescents to be able to develop, and that strengthening the existing smoking rules with prohibitions to leave the school premises will cause relational difficulties. Firstly, smokers will “find other ways” (4T) to “smoke in secrecy” (2S) and thereby it becomes more difficult for staff members and smokers to form a personal and honest bond. Secondly, prohibiting the addicted smokers from smoking will decrease their ability to “follow classes for three hours straight” (1ST), make them feel “less excited to come to school” and so “there’ll be little that they can learn” (4T). Lastly, more strict rules likely lead to continuous conflicts between smokers and staff members because it would be a radical measure to prohibit everyone from smoking for a full day. Avoiding these difficulties was considered particularly important for adolescents growing up in “home environments with little support” (4S) so that the schools can be a place for them where they feel at home and safe.

Smoking during school hours was accordingly framed as something that some adolescents need—that is, something that they cannot live without and hence schools should have to take this into account.

I just think that we as a school will never be that strict. In this school we have very clear rules, but we also try to take into account the individual [different] situations and needs. And when taking into account these situations and needs, so if there are pupils who smoke, it is just in ‘our system’ to always look for a solution. That happens with pupils who need extra support and with the other pupils as well, and in this case also with the smoking policy (1T).

So rather than “forcing everything [related to smoke] through rules” (2T), staff members argued that schools should focus on convincing adolescents not to smoke by staying in dialogue with the smokers, talking with them about why they smoke, and educating them about the consequences.

In order to achieve anything, it should be possible to talk with each other, to communicate. You should achieve something together. So if the only thing you’re doing is challenging each other [about smoking rules], then I believe you’ll never achieve something (1S).

Yes, and it’ll [strict smoking rules] work against you. You shouldn’t want it. (…) The more you patronize them, the less you’ll be able to do with them, because then you’re no longer by their side, but you’re constantly in conflict about what is and isn’t allowed (4S).

### Secondary Belief: Schools Should Only Adopt Rules That They Are Able to Consistently Enforce

The staff members were reluctant to adopt rules that would make school hours a smoke-free period because “you have to be able to enforce rules once you establish them” (1P). This is a prerequisite for adoption because adolescents need clarity about what is and is not allowed; adolescents will make use of ambiguities and therewith undermine schools’ authority over adolescent smoking behavior.

If you create a rule, you should be able to enforce it. Else it’ll become quite difficult and you’ll continually get into discussions (4P).

Staff members mentioned two main issues that would make it difficult to consistently enforce smoke-free school hours. First, schools would need to spend more time enforcing the rules than currently is the case because the school premises oftentimes are not closed off from outside (e.g., by a fence) and students have individual schedules, starting and ending their schooldays at different times.

If I would like to make this rule any more strict, which means no one is allowed to smoke on school grounds anymore, and no one is allowed to leave school grounds anymore, and I would like to enforce that, I would have to station people all over the place during each break. They will have to check whether anyone would want to leave school grounds. ‘Show me your student card, let’s see your school schedule’, that’s just not realistic (2P).

Well, we can put strict rules in place and that requires a lot of other things from us. The students will find other ways and look for things like that and then we’ll constantly be busy with that, and now we all have it reasonably under control (4T).

Second, smoke-free school policies would require the contribution of all staff members. This was considered to be problematic as in some schools there are teachers who even resist to contribute to the currently less strict rules.

They won’t do that. No, they’ll yell that ‘I need my break (3S).

Smoking teachers at some schools, moreover, actively resisted schools’ plans to become more strict. One smoking teacher mentioned how he would undermine school authority if it was decided that he was not allowed to smoke in his current shed anymore.

We’ll [smoking teachers] be standing on the streets so we won’t be breaking any rules, but it does mean that all students will be able to see us [smoke to provoke management] (2T).

One senior teacher made a parallel with existing rules on wearing caps in classes to underpin the need for consistency and why all staff members’ contribution is so important.

Wearing caps during class is officially not allowed, and the moment that every teacher consistently enforces that rule, then within a week you won’t see anybody wearing caps in class anymore. If there are two or three teachers who deviate from that, then you can be 100% sure that there will be a student who says: ‘Well, this other teacher does allow it.’ As a result, you won’t be able to enforce the rule. So the core is to have clearly established agreements, and to consistently enforce these as a team of teachers (1ST).

## Discussion

Drawing on the Advocacy Coalition Framework, we identified school staff member’s deep core, policy core, and secondary beliefs about the adoption of rules that would prohibit adolescents from leaving the school premises to establish smoke-free school hours.

Two deep core beliefs were identified: (i) schools should provide adolescents the freedom to learn how to make responsible use of their personal autonomy and (ii) schools should interfere if adolescents endanger or bother others. Three policy core beliefs were identified: (iii) smoking is not a pressing issue for schools to deal with (iv) schools should demarcate their jurisdiction to intervene in adolescents’ lives in time, space, and precise risk behavior (v) implementing smoke-free school hours would interfere with maintaining positive student-staff relationships. The one secondary belief was that (vi) smoke-free school hours would be impossible to enforce consistently.

### Limitations

There are three limitations that should be taken into account when interpreting the findings. First, we used data that was collected only in one city. Therefore, generalization of these precise beliefs to other schools in the Netherlands, and even more so in Europe, should be done with caution.

Second, our sample size did not allow us to systematically compare between the different school levels or staff functions. We cannot exclude that the beliefs may have somewhat different nuances for staff members in higher versus lower educational schools, or between those in management versus supportive functions.

Lastly, we did not stop data collection upon reaching theoretical saturation during analysis, as our secondary analysis was performed on existing data. Doubts may thus remain about whether we have missed some nuances that would have further enriched the six identified beliefs.

### Interpretation of Results

The two deep core beliefs build strongly on the Western connotation of personal autonomy: individuals should have freedom over their choices as long as this choice does not violate others’ freedoms. Staff members’ emphasis on adolescents’ autonomy, even if this leads to unhealthy choices, corresponds with evidence that Dutch culture is highly individualistic [25]. This importance of individual choice and own responsibility was also found among 15–16-year old Dutch adolescents, who—including non-smokers—were shown to oppose the adoption of new tobacco control policies that seem to conflict with smokers’ right to smoke [24]. Therefore, it is not surprising to encounter strong resistance against a policy that would constrain the autonomy of all adolescents to leave the school premises.

Staff members made a distinction between younger and older adolescents when reasoning about schools’ current rules on restricting the autonomy of adolescents. Younger adolescents were reasoned to need protection against possible unhealthy choices, whereas older adolescents needed to be granted freedom of choice to develop into autonomous adults. The choice to protect the younger adolescents corresponds with an influential Dutch governmental advisory report stating that minors need protection against their autonomy because they are insufficiently able to oversee the consequences of unhealthy behavior and are insufficiently capable to resist unhealthy temptations that they encounter [26]. However, it remains difficult to explain why staff members deviate from the advisory report (i.e., minors are below the age of 18) and already consider adolescents from 14/15 years onwards to be sufficiently able to oversee consequences and resist temptations. The two most likely explanations are that the law on the age of tobacco sales has only recently increased from 16 to 18 years (i.e., in 2014) as well as that in earlier times much less was known about the process of neurocognitive development that lasts until a person is approximately 25 years of age [33].

Two policy core beliefs were that smoking per se is not a pressing issue and that schools should demarcate their jurisdiction to intervene in someone’s smoking. These beliefs are interesting given that schools’ approaches to the use of alcohol and drugs are much more strict (i.e., prohibited at all times during school hours) than how they deal with smoking. This discrepancy indicates that school staff members in the Netherlands still consider adolescent smoking as a relatively acceptable behavior.

The third policy core belief was that staff members should be able to maintain positive relationships, particularly with the most vulnerable smokers, and that prohibiting them from smoking outside the premises will damage these relationships. A similar prioritization of staff-student relationship over tackling smoking was reported for Scottish teachers [29]. These concerns may relate to the limited effective support that schools can offer to smoking adolescents; there is no robust evidence about effective cessation interventions for adolescents [34]. This implies that the only option for staff members to enforce smoke-free school hours would be to sanction the vulnerable adolescents who smoke during school hours—an approach that challenges staff members’ role perception and what they believe is most beneficent for the students’ well-being and academic performance [29].

The secondary belief was that far-reaching policies lead to an ever-contested, personally demotivating, and time-consuming policy to enforce and the associated fear that this would lead to inconsistencies in staff members’ enforcement practices. Prior research suggests that inconsistent enforcement decreases adolescents’ acceptance of the school authority over their smoking and provides adolescents the opportunity to collectively rebel against the school rules [8, 29]. Some of the earlier identified barriers for the adoption of SFSPs, such as high workload, open premises, and resistance from smoking staff [12], may thus partly explain staff members’ concern to lose control and authority over adolescents’ behaviors. Overall, the findings demonstrate that studying stakeholders’ deep core, policy core, and secondary beliefs about the implementation of health policies may provide a complementary perspective to existing studies that aimed to understand their (non-)adoption. Studies commonly explored the facilitators and barriers of implementation or assessed key predictors of implementation, but hardly took into account the deep-rooted values, rationales, and assumptions that hinder the implementation of health policies by those with decision-making power. We therefore suggest the application of a similar approach to understand the belief systems of those with decision-making power for other health promoting policies (e.g., prohibition to sell soft drinks) and in other organizations (e.g., businesses). Key to this approach is that the researcher teases out the tensions between what is and what is not implemented. Like in our case, what explains why school staff members are fine to prohibit adolescents until the third grade from smoking, but suddenly becomes problematic when an adolescent reaches the fourth grade.

### Practical Implications

The ACF posits that stakeholders’ beliefs may change due to exposure to new or better information that contests the currently held beliefs [22]. While more evidence and discussion about the effectiveness and desirability is needed, we provide four brief suggestions on how to convince school staff to gradually make school hours a smoke-free period for adolescents of increasingly higher age.

Firstly, advocacy efforts may aim to take advantage of staff’s view that personal autonomy is a core value, by emphasizing that nicotine dependence decreases the ability to choose to smoke or not to smoke. The decision to start or continue smoking during school hours may even have lifetime consequences on individuals’ autonomy due to the addictive nature of nicotine. Smoke-free school hours that aim to further decrease adolescent smoking may therefore be presented as a potential means to increase long-term personal autonomy.

Secondly, advocacy efforts may take advantage of staff’s aspiration to teach adolescents how to make responsible use of their personal autonomy, by presenting smoke-free school hours as the structure that adolescents need to effectively do so. Neuroscientists increasingly argue that adolescents’ brains are not mature enough to resist external stimuli (e.g., cigarettes, unhealthy food) and make considered choices [33]. Schools providing adolescents 14/15 years of age too much freedom may therefore actually all but support them [35].

Thirdly, advocacy efforts may aim to make clear that adolescent smoking during school hours is a more pressing issue than it currently seems to be. One strategy could be, like the Swedish Teachers against Tobacco initiative [36], to unite likeminded staff members and support them in advocating for smoke-free school hours within their respective schools. Key advocacy arguments could be that smoking influences academic achievement [37] and that seeing older adolescents smoke outside the premises increases younger adolescents’ likelihood to initiate smoking [6, 7].

Lastly, we urge other stakeholders to develop and/or identify effective strategies for schools to support nicotine-dependent adolescents to quit smoking, so that staff members can enforce the smoking rules by offering rule violators help instead of giving punitive sanctions. Punitive sanctions may indeed have a negative influence on the relationship between the punisher and the offender [38] and even an adverse impact on the smoking behaviors of the most vulnerable adolescents [39].

## Conclusions

Drawing on the Advocacy Coalition Framework, this paper was the first to demonstrate the deep core, policy core, and secondary beliefs explaining why school staff members refrain from implementing far-reaching smoke-free policies. The new insight derived from this approach could be used to determine how school staff can be convinced to gradually make school hours a smoke-free period for adolescents of increasingly higher age.
